# Sperm Chromatin Dispersion Test before Sperm Preparation Is Predictive of Clinical Pregnancy in Cases of Unexplained Infertility Treated with Intrauterine Insemination and Induction with Clomiphene Citrate

**DOI:** 10.3389/fmed.2016.00063

**Published:** 2016-11-23

**Authors:** Frank W. R. C. Vandekerckhove, Ilse De Croo, Jan Gerris, Etienne Vanden Abbeel, Petra De Sutter

**Affiliations:** ^1^Centre for Reproductive Medicine, University Hospital, Ghent, Belgium

**Keywords:** DNA fragmentation, chromatin dispersion test, oxygen radicals, *in vitro* fertilization, receiver operating characteristic

## Abstract

**Background/aims:**

A large proportion of men with normal sperm results as analyzed using conventional techniques have fragmented DNA in their spermatozoa. We performed a prospective study to examine the incidence of DNA fragmentation in sperm in cases of couples with previously unexplained infertility and treated with intrauterine insemination. We evaluated whether there was any predictive value of DNA fragmentation for pregnancy outcome in such couples.

**Methods:**

The percentage of DNA fragmentation and all classical variables to evaluate sperm before and after sperm treatment were determined. We studied the probable association between these results and pregnancy outcome in terms of clinical and ongoing pregnancy rate per started first cycle. We also assessed the optimal threshold level to diagnose DNA fragmentation in our center.

**Results:**

When using threshold levels of 20, 25, and 30%, the occurrence of DNA fragmentation was 42.9, 33.3, and 28.6%, respectively. Receiver operating characteristic (ROC) analysis of all cases revealed an area under the curve of 80% to predict the clinical pregnancy rate per cycle from testing the sperm motility (a + b) before treatment. We failed to generate an ROC curve to estimate pregnancy outcome from the amount of DNA fragmentation before treatment. However, when selecting only those men with a pretreatment DNA fragmentation of at least 20%, the pretreatment result was statistically different between couples who achieved a clinical pregnancy and those who did not.

**Conclusion:**

DNA fragmentation is often diagnosed in couples with unexplained infertility. Each center should evaluate the type of test it uses to detect DNA fragmentation in sperm and determine its own threshold values.

## Introduction

Recent research has revealed that subtle abnormalities can be found in sperm samples that seem to be normal according to conventional analysis techniques (1, 2). The DNA in the sperm head is sometimes fragmented, and this may be the reason why couples with a diagnosis of unexplained infertility do not achieve pregnancy. There seems to be a correlation between sperm fragmentation and aneuploidy (3).

DNA damage in spermatozoa affects both mitochondrial and nuclear DNA (4). Its origin can be explained by six main mechanisms:

– apoptosis during the process of spermatogenesis;
– DNA strand breaks that occur during the remodeling of sperm chromatin in spermatogenesis;
– post-testicular DNA fragmentation induced mainly by oxygen radicals (ROS), including the hydroxyl radical and nitric oxide, during sperm transport through the seminiferous tubules and the epididymis; the effect of ROS on sperm has been known since 1943 (5);
– DNA fragmentation induced by endogenous caspases and endonucleases;
– DNA damage induced by radiotherapy and chemotherapy; and
– DNA damage induced by environmental toxicants.

Fifteen percent of men with normal basic semen analysis profiles (6) have been associated with infertility problems (7). Moreover, about 8% of men with normal sperm results do have abnormal levels of DNA fragmentation in sperm (8).

Most studies of prognostic factors for pregnancy with intrauterine inseminations (IUI) focus on the motility of the sperm after treatment. A total motile count of 1–2 million sperm cells is usually regarded as a threshold value to obtain a pregnancy in cases of unexplained or mild male infertility (9). More recent studies have provided evidence for the inverse relationship between clinical outcomes of IUI and the amount of DNA fragmentation (8).

We planned a prospective observational study to examine the incidence of DNA fragmentation in sperm in cases of couples with previously unexplained infertility. We assumed that DNA fragmentation was likely to be present in sperm for a number of couples with the so-called unexplained infertility. We also selected this group of patients to exclude as many confounding diagnostic factors as possible. In the first treatment cycle with IUI, the percentage of DNA fragmentation in sperm was measured before and after sperm preparation. We evaluated if there was any predictive value of DNA fragmentation for pregnancy outcome. Afterward, the results of pregnant patients were compared with those who were not, for different threshold levels of DNA fragmentation.

## Materials and Methods

This prospective study was approved by the Ethical Committee of Ghent University Hospital and internationally (http://ClinicalTrials.gov approval number: NCT02235103). Between March 1, 2014 and February 1, 2016, we recruited 25 patients. Study enrollment depended on the availability of laboratory facilities on a daily basis. A written and informed consent was obtained from all participants.

We included only first treatment cycles of couples with unexplained infertility. Female patients were checked for tubal patency by hysterosalpingography or hysterosalpingo-foam sonography (10, 11) or by laparoscopy if indicated. Their cycles were documented as ovulatory. Uterine and ovarian abnormalities were excluded by clinical and ultrasound examination. All the included female patients were between 18 and 40 years old.

A sperm analysis was performed before treatment and revealed no abnormalities according to the WHO criteria (12). Diagnostic sperm analysis was carried out using the automated SCA^®^ system (Sperm Class Analyser, CASA System, Microptics, Barcelona, Spain). Morphology assessment was done manually using Spermblue^®^ staining (Microptics).

According to our protocol and evidence-based guidelines (13), the patients were treated by ovarian stimulation (50-mg clomiphene citrate starting from cycle day 3, for five consecutive days) and IUI. Monitoring was performed *lege artis* (14).

**Figure 1:**
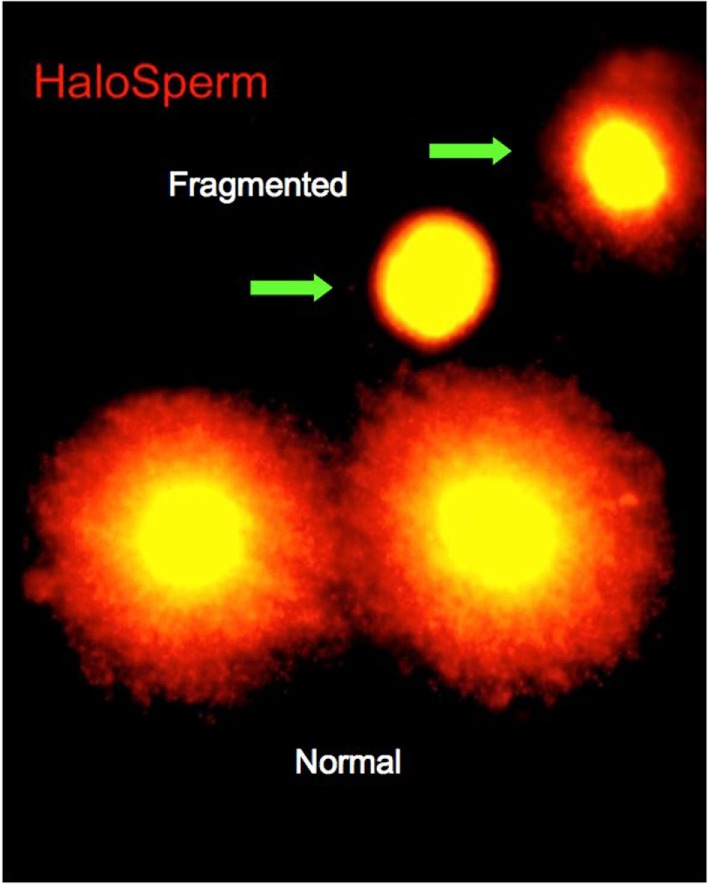
**Interpretation of the Halosperm test**.

On the day of treatment, semen specimens were collected by masturbation into sterile cups. Semen was allowed to liquefy for 30 min, and an aliquot was taken for macroscopic and microscopic assessments. Specimens were assessed for volume, count, motility, and morphology. The first assessment of DNA fragmentation was carried out at this time. Of the various tests currently available for determining DNA fragmentation, we selected the sperm chromatin dispersion (SCD) test (Halosperm^®^; Halotech, Madrid, Spain). The SCD test assesses the capacity of the sperm chromatin to disperse, under the effect of hydrochloric acid to denature the chromatin, and to generate restricted single-stranded DNA motifs from DNA. After denaturing, a lysing solution was used, and the level of DNA fragmentation was estimated by the size of the nuclear dispersion and measured using immunofluorescence or optical microscopy (Figure 1). The amount of dispersion is inversely proportional to the level of DNA damage (8).

Sperm preparation was performed using a two-layer (90 and 45%) percoll gradient supplied by Cook^®^ (Cook, Limerick, Ireland) with centrifugation at 400 × *g* for 20 min. The procedure is described in more detail elsewhere (15). After this capacitation procedure, a diagnostic sperm evaluation, except for the morphology assessment and the SCD assay, was repeated on an aliquot. IUI followed without delay because shortening the time period between semen processing and insemination minimizes sperm DNA fragmentation (4).

The outcome variables studied were the clinical pregnancy rate and the ongoing pregnancy rate per started cycle as defined by the WHO criteria (16).

We used receiver operating characteristic (ROC) curve analysis for testing the sensitivity and specificity of different sperm variables in predicting the pregnancy outcome. Sperm concentration, motility, and morphology, as well as DNA fragmentation before and after sperm preparation and the percentage amelioration (difference) of DNA fragmentation after sperm preparation, were included in the analysis.

Filtering the data for different cut-off levels of sperm DNA fragmentation allowed us to see if there were any differences in sperm variables between the pregnant and non-pregnant groups in these different cohorts. All analyses were performed for clinical and ongoing pregnancies as outcome variables. A cut-off level of 30% is usually cited in the literature for the SCD assay (17). We used 20, 25, and 30% as cut-off levels for our analysis.

Statistical analysis was performed using the SPSS V23. Fisher’s exact test was applied for proportions and the non-parametric Mann–Whitney *U* test for continuous outcomes. *p* Values <0.05 were considered statistically significant.

## Results

Although 25 patients were originally intended for inclusion, only 21 could be retained for further analysis. Three patients were excluded because of a lack of weekend laboratory facilities, and one was excluded because of missing data regarding the male history.

**Figure 2:**
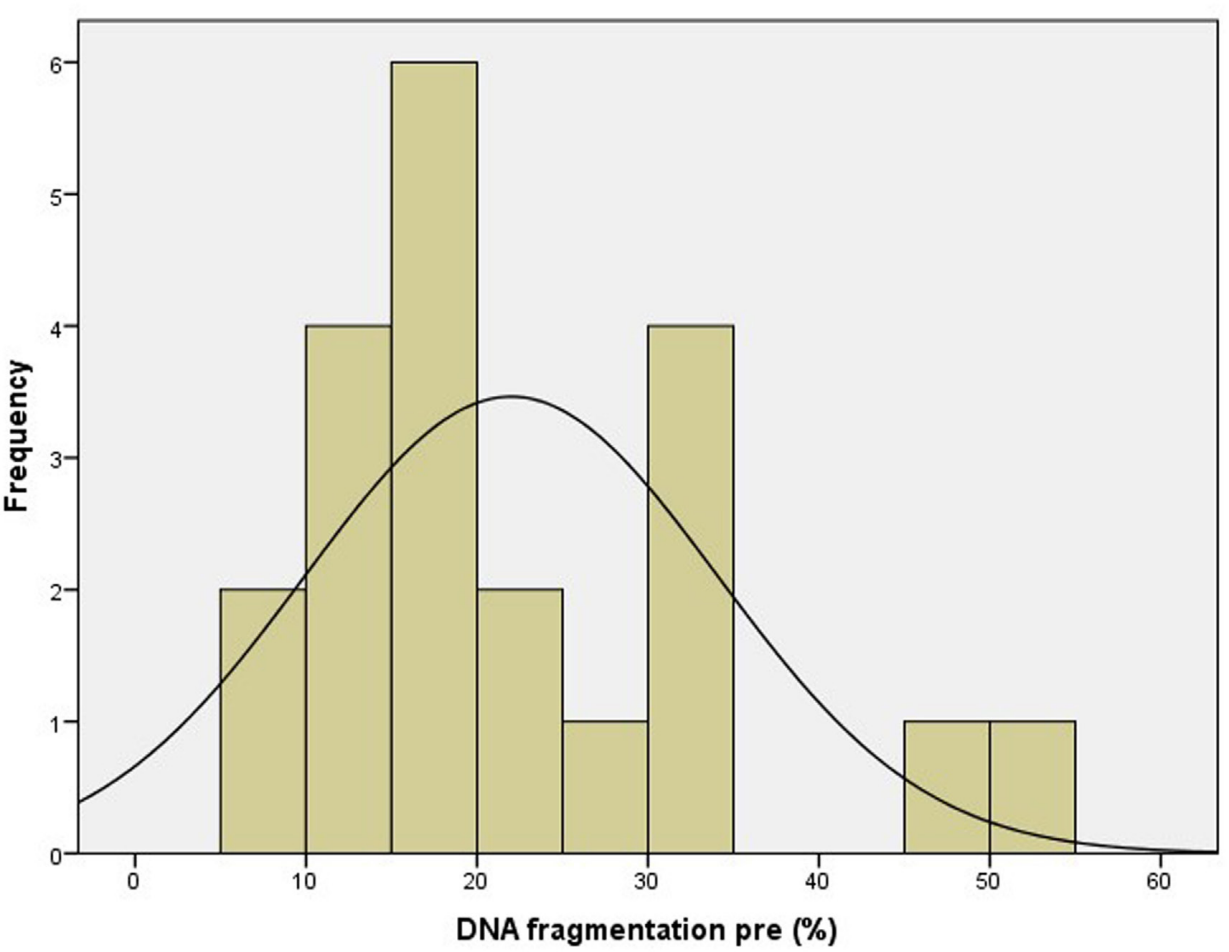
**Distribution curve of DNA fragmentation before treatment**.

Patient characteristics are summarized in Table 1. Only cases of unexplained male and female subfertility were included, as illustrated by normal values for the diagnostic sperm analysis. The distribution curve of the values obtained with the pretreatment DNA fragmentation test is illustrated in Figure 2. Nine patients (42.9%) showed an abnormal level of DNA fragmentation at a cut-off level of 20%. At the level of 25 and 30%, the numbers were 7 (33.3%) and 6 (28.6%), respectively.

**Table 1:** **Descriptive statistics**.

Variable	
Number of patients	21
Female age (years)	33.8 (4.1)
Female body mass index (kg/m^2^)	23.0 (4.2)
Duration of infertility (years)	2.2 (1.3)
Anti-Müllerian hormone (ng/mL)	2.5 (1.5)
Male age (years)	35.8 (8.2)
Male body mass index (kg/m^2^)	25.7 (3.6)
Sperm concentration (×10^6^/mL)	72.7 (40.5)
Sperm motility a + b (%)	52.8 (13.5)
Normal sperm morphology (%)	6.8 (3.8)
Sperm motility pre a + b (%)	46.1 (17.4)
Total motile count pre (×10^6^)	86.3 (62.2)
Normal sperm morphology pre (%)	8.0 (3.9)
DNA fragmentation pre (%)	22.0 (12.1)
Total motile count post (×10^6^)	27.9 (23.8)
DNA fragmentation post (%)	6.3 (5.9)
DNA fragmentation difference (%)	68.5 (35.7)
Clinical pregnancy rate per cycle (%)	23.8
Ongoing pregnancy rate per cycle (%)	19.0

*Numbers are expressed as means (SD) unless explained differently*.

The results of DNA fragmentation tests before and after treatment, as well as the DNA fragmentation differences for individual patients, are summarized in Table 2.

**Table 2:** **Individual data on DNA fragmentation**.

Patient number	DNA fragmentation pre (%)	DNA fragmentation post (%)	DNA fragmentation difference (%)
1	50.0	10.5	79.0
2	22.5	22.0	2.2
3	18.0	1.5	91.7
4	33.5	15.5	53.7
5	30.0	4.5	85.0
6	30.0	3.5	88.3
7	8.5	5.0	41.2
8	15.0	0.0	100.0
9	12.0	3.5	70.8
10	21.5	5.5	74.4
11	8.5	13.0	−52.9
12	34.0	15.0	55.9
13	12.0	2.0	83.3
14	15.0	1.0	93.3
15	10.0	1.0	90.0
16	28.0	7.5	73.2
17	49.0	9.0	81.6
18	14.0	4.5	67.9
19	15.0	0.5	96.7
20	17.5	1.5	91.4
21	18.0	5.0	72.2

Receiver operating characteristic curve analysis was performed for the following sperm variables to test their specificity and sensitivity in predicting clinical pregnancy rate and ongoing pregnancy rate per started cycle, respectively:

– sperm concentration, motility (a + b), and normal morphology in the diagnostic sample;
– motility (a + b), total motile count, normal morphology, and DNA fragmentation in the native sample before capacitation;
– motility (a + b), total motile count, and DNA fragmentation in the treated sample; and
– percentage of amelioration of DNA fragmentation in the treated sample.

**Figure 3:**
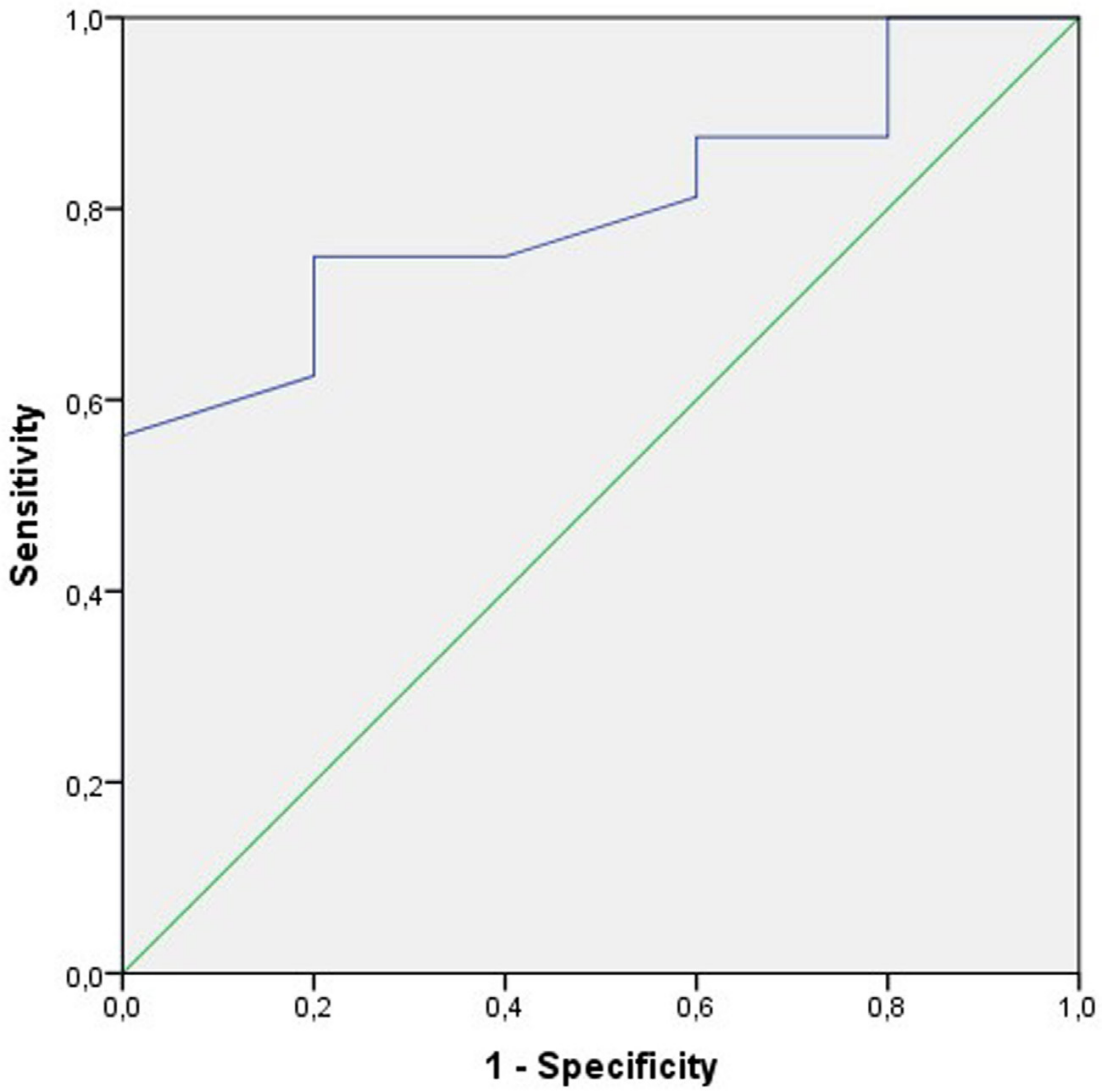
**Receiver operating characteristic (ROC) curve to test the sensitivity and the specificity of the sperm motility a + b before treatment to predict the clinical pregnancy rate per cycle**. The area under the curve (AUC) is 80% (95% CI: 61–99%); *p* < 0.05.

The ROC curve on motility (a + b) before treatment to estimate the probability of clinical pregnancy per treatment cycle was the only parameter that satisfied the statistical requirements. The area under the curve AUC was 80% [95% confidence interval (CI): 61–99%; *p* < 0.05] (Figure 3).

None of the DNA fragmentation variables fulfilled the statistical criteria to be considered useful. Either the AUC was too low (<70%) or the association was insignificant (*p* ≥ 0.05).

**Figure 4:**
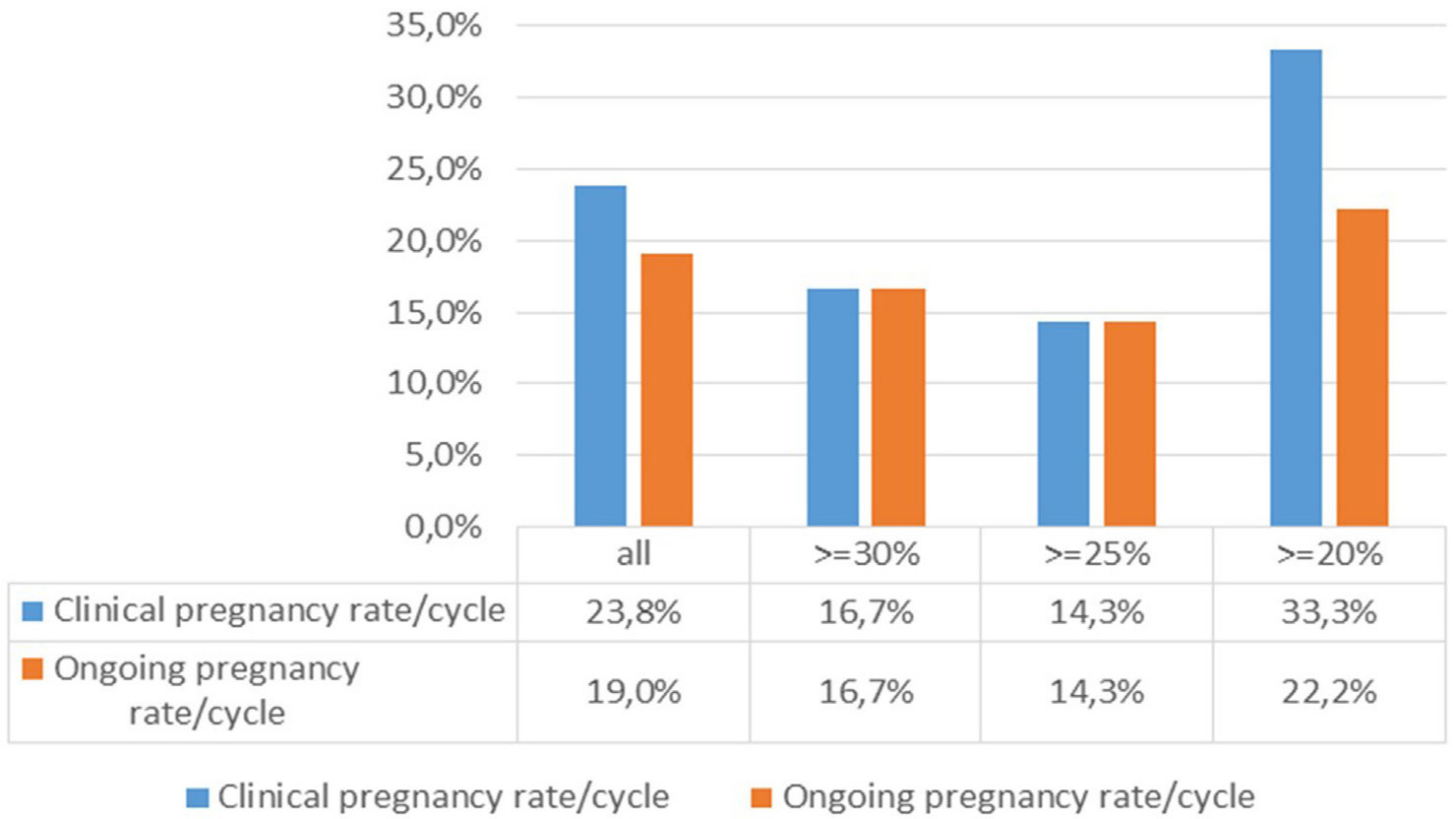
**Association between the pregnancy rates per cycle and the degree of DNA fragmentation pretreatment**.

The association between the pregnancy rates per cycle and the degree of pretreatment DNA fragmentation (Figure 4) was analysed by calculating the clinical and ongoing pregnancy rates per started cycle for different patient cohorts. When filtering the results for patients with minimum levels of 20, 25, and 30% DNA fragmentation, respectively, the difference between the ≥20% group and those with higher levels of DNA fragmentation was striking, although not statistically significant (Fisher’s exact test).

**Figure 5:**
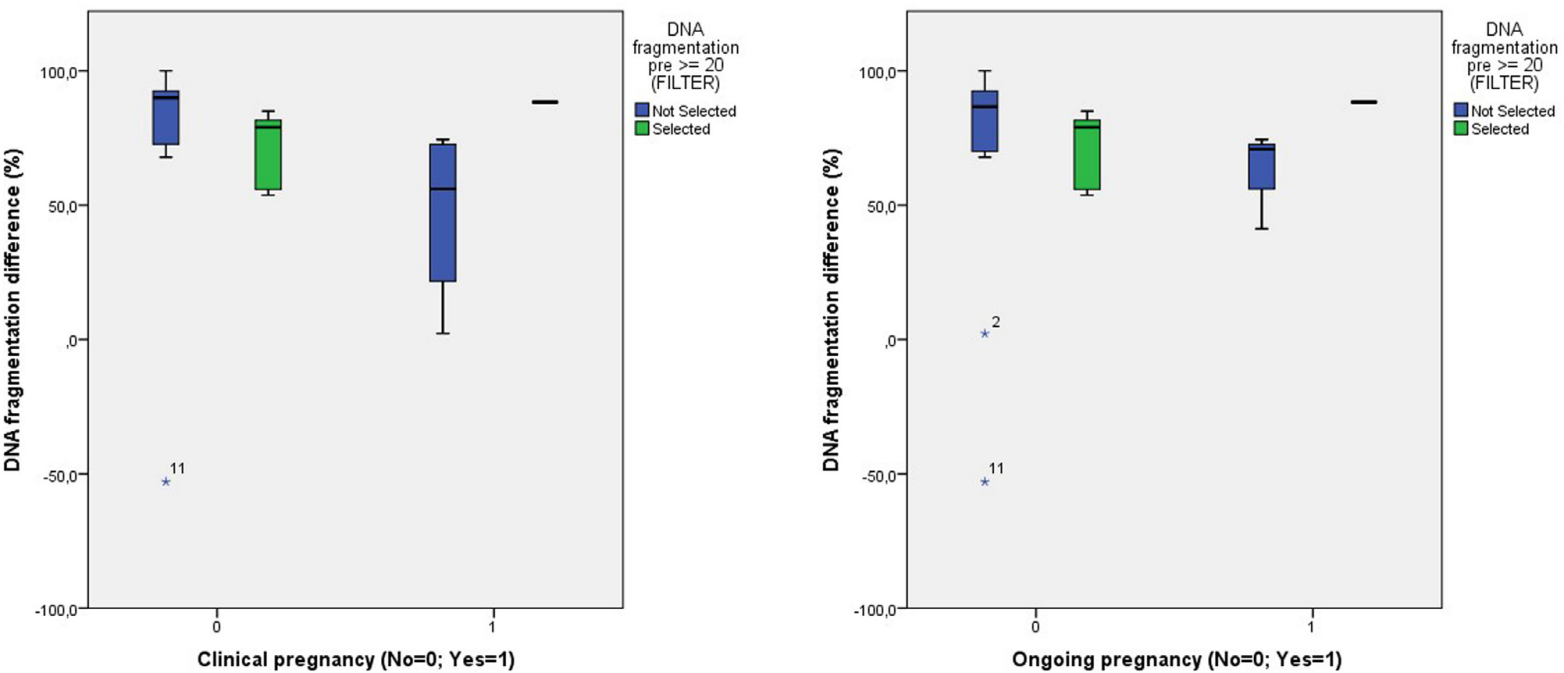
**Comparison of the degree of DNA fragmentation difference and the clinical and ongoing pregnancy outcome between all patients and those with a pretreatment DNA fragmentation of ≥20%**.

When comparing the degree of amelioration of DNA fragmentation between the entire patient group and those with a pretreatment level of DNA fragmentation of ≥20%, again, we found no differences in pregnancy rates (Figure 5).

After filtering the data for patients with a pretreatment DNA fragmentation of 20%, all previously mentioned sperm variables were analyzed for equality between the pregnant and non-pregnant patients, both for clinical and ongoing pregnancies. Because of the unequal variances, a Mann–Whitney *U* test was performed. The same evaluation was performed after filtering data for patients with a pretreatment DNA fragmentation value of ≥25 and ≥30%.

**Figure 6:**
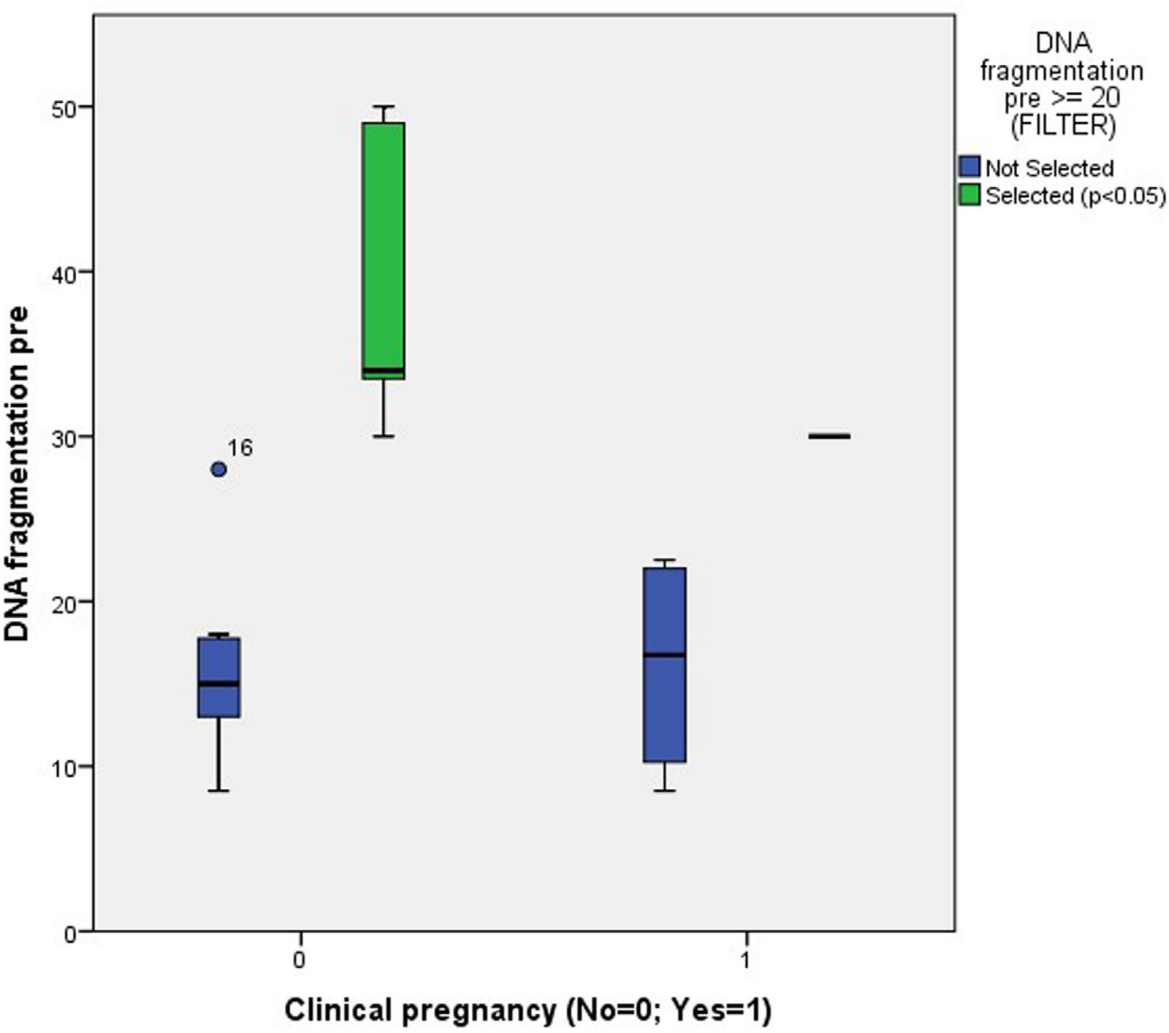
**Association between clinical pregnancy and the degree of pretreatment DNA fragmentation in unselected patients (NS) and patients with a pretreatment DNA fragmentation of ≥20% (*p* < 0.05)**.

The only significant finding was a difference in the percentage of pretreatment DNA fragmentation between patients who were clinically pregnant vs. those who were not and who already had a pretreatment value of ≥20% (Figure 6).

## Discussion

Various assays have been developed to measure either the proportion of spermatozoa displaying DNA fragmentation in a sperm sample or the DNA damage per spermatozoon (8). Examples of the former are the sperm chromatin structure assay (SCSA), SCD, and terminal deoxynucleotidyl transferase-mediated dUTP nick-end labeling (TUNEL) tests. The Comet test (single-cell gel electrophoresis) belongs to the latter category. Despite their variability in protocol, comparative studies have shown close correlations between DNA damage measured by these different commonly used assays (7). Our choice for SCD was made from a practical point of view: simplicity, cost-effectiveness, reproducibility, and lack of requirement for special equipment.

The debate on the routine use of testing for the degree of DNA fragmentation in sperm is ongoing (18). Nowadays, most reports provide evidence for a negative impact of elevated DNA fragmentation on the clinical outcome of treating couples by means of timed intercourse or IUI (8). However, its relevance in *in vitro* fertilization (IVF) and intra-cytoplasmic sperm injection (ICSI) remains more elusive. A recent meta-analysis revealed a higher live birth rate in IVF-treated couples with low DNA fragmentation; a sensitivity analysis showed no difference when ICSI was used (19). The type of assay may also be important. When focusing on an SCD test, such as the one we performed, a prospective study showed no association with embryological data or pregnancy rates (20). The Comet test, on the other hand, seemed to have a predictive value for IVF outcome (21). The fact that the results of Assisted Reproductive Technology (ART) treatment by IVF or ICSI were less influenced by the degree of DNA fragmentation can be explained by the fact that ovarian hyperstimulation as used in IVF and ICSI provided a higher number of oocytes. Consequently, there was a better recruitment of good-quality oocytes with an intact repair mechanism after they were fertilized by DNA-defective sperm (4, 22). Most authors formulated a call for more robust studies (23).

We included patients with unexplained male and female infertility only in order to exclude as many confounding factors as possible. This is likely to be the reason why we detected such a large number of cases with elevated DNA fragmentation. As other factors were excluded, probably the DNA fragmentation itself was an explanation for the reproductive failure.

A major flaw in reviews of DNA fragmentation is the use of different thresholds for DNA damage (24). Most reports about assays using chromatin structure used cut-off levels of 27–30% (25). The same authors even changed their values between one study and another (17, 26). Therefore, we refined our analysis by using different cut-off levels. There is strong evidence from our center that a cut-off level of 20% is the most predictive value. Therefore, we suggest that fertility units should adhere to a single type of test and analyze their data to obtain an individual threshold value.

Although DNA fragmentation testing is performed on whole sperm, some authors have suggested analyzing only motile sperm (8, 27) or morphologically normal sperm (22). Most authors agree that dead sperm cells in a sample can influence the results from most types of assay (8).

Several authors have confirmed a “healing” effect of sperm preparation on the amount of DNA fragmentation (21, 27). However, the procedure either did not completely remove the parts of the DNA that were fragmented part (22) or else it had a negative effect as shown in an isolated case in our study (case 11 in Table 2).

There are some challenges that need to be addressed in future studies. A damaged spermatozoon can fertilize an oocyte, and a conceptus with suboptimal paternal integrity may develop (8). Hence, further research to develop technological methods for the selection of individual DNA-intact sperm cells should continue (22).

Because our threshold level when using DNA fragmentation differs from those of earlier studies (17, 25, 26), we suggest that each center should calculate its own cut-off levels. Our study can be used as a pilot template for this purpose.

## Author Contributions

FV wrote the manuscript; FV and IC collected the data and performed the analysis; JG, EA, and PS critically reviewed the manuscript.

## Conflict of Interest Statement

The authors declare that the research was conducted in the absence of any commercial or financial relationships that could be construed as a potential conflict of interest.
